# Covid-19 imaging: A narrative review

**DOI:** 10.1016/j.amsu.2021.102489

**Published:** 2021-06-18

**Authors:** Hanae Ramdani, Nazik Allali, Latifa Chat, Siham El Haddad

**Affiliations:** Radiology Department, Childrens' Hospital - Ibn Sina University Hospital-Rabat, Lamfadel Cherkaoui Street, 10010, Rabat, Morocco

**Keywords:** COVID-19, Sars-Cov2, Novel coronavirus, Imaging, Computed tomography (CT), Chest

## Abstract

**Background:**

The 2019 novel coronavirus disease (COVID-19) imaging data is dispersed in numerous publications. A cohesive literature review is to be assembled.

**Objective:**

To summarize the existing literature on Covid-19 pneumonia imaging including precautionary measures for radiology departments, Chest CT's role in diagnosis and management, imaging findings of Covid-19 patients including children and pregnant women, artificial intelligence applications and practical recommendations.

**Methods:**

A systematic literature search of PubMed/med line electronic databases.

**Results:**

The radiology department's staff is on the front line of the novel coronavirus outbreak. Strict adherence to precautionary measures is the main defense against infection's spread. Although nucleic acid testing is Covid-19's pneumonia diagnosis gold standard; kits shortage and low sensitivity led to the implementation of the highly sensitive chest computed tomography amidst initial diagnostic tools. Initial Covid-19 CT features comprise bilateral, peripheral or posterior, multilobar ground-glass opacities, predominantly in the lower lobes. Consolidations superimposed on ground-glass opacifications are found in few cases, preponderantly in the elderly. In later disease stages, GGO transformation into multifocal consolidations, thickened interlobular and intralobular lines, crazy paving, traction bronchiectasis, pleural thickening, and subpleural bands are reported. Standardized CT reporting is recommended to guide radiologists. While lung ultrasound, pulmonary MRI, and PET CT are not Covid-19 pneumonia's first-line investigative diagnostic modalities, their characteristic findings and clinical value are outlined. Artificial intelligence's role in strengthening available imaging tools is discussed.

**Conclusion:**

This review offers an exhaustive analysis of the current literature on imaging role and findings in COVID-19 pneumonia.

## Highlights

• Chest computed tomography is a highly sensitive Covid −19 pneumonia's diagnostic tool.
• Initial Covid-19 CT features are bilateral, multifocal, peripheral or posterior ground-glass opacities, mainly in the lower lobes.
• Multifocal consolidations, bronchiectasis, pleural thickening, and subpleural bands are late disease stages features.
• Standardized CT reporting is recommended to guide radiologists.
• Artificial intelligence could strengthen available imaging tools.

## Graphical abstract

**Figure:**
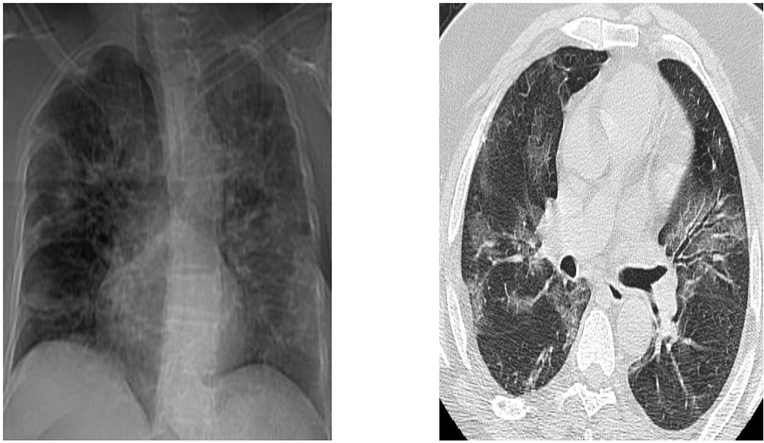

## 1 Introduction

The emerging Covid-19 pandemic continues its rapid spread challenging healthcare systems worldwide in a new and unpredictable manner. Communication and shared experience are key elements to better understand the infection and thus control it through rapid diagnosis, early quarantine and prompt treatment [1]. In the face of the extremely communicable SARS-COV2 outbreak, continual assessment of the highly-dynamic available data, further large systematic analyses and prospective observational and clinical trials are essential to protect communities and healthcare personnel, prepare human capital, arrange infrastructure, effectively manage patients and surveil public health [2,3]. Alongside with epidemiological history, clinical characteristics and laboratory findings; imaging plays an invaluable role in early recognition, triage, prognosis prediction and may be of use in therapeutic evaluation and follow-up of Covid-19 pneumonia [[4], [5], [6]]. This report summarizes precautionary measures for radiology departments whose staff is on the front line of this alarming outbreak, Chest CT's role in diagnosis and management, imaging findings of COVID-19 patients including children and pregnant women, artificial intelligence applications in this setting and practical recommendations.

## 2 Radiology department organization

Radiology departments' strict adherence to robust protective protocols is mandatory to minimize virus spread via droplets transmission and contaminated equipment [7,8]. Non urgent exams are rescheduled while hospitalized and urgent external patients' studies (malignancies in particular) are performed [9,10]. On-site staff is reduced, rotations are established and the largest possible number of functionaries work from home. Deployment in high-priority areas is a possibility to prepare for [9,10]. Training programs are suspended [10]. Social distancing is implemented. Videoconferencing and telephone are utilized whenever practicable [9,10]. Employees are taught how to appropriately use Personal protective equipment (PPE) with emphasise on the necessity of regular and thorough washing of hands [9]. Wearing a mask (surgical or rather a respirator), eye protection (goggles or face shield), an isolation disposable gown and gloves is recommended when in close contact with suspected or confirmed Covid-19 patients. Shoe covers and a surgical cap could be added [[7], [8], [9]]. When possible, portable imaging limits suspected patients' movement [8]. If transportation to the radiology unit is needed, patients are to take a well-delineated path and wear a surgical mask throughout transfer and examination [7,9]. Dedicated CT scanners reduce contamination and allow high throughput as the needed exam (unenhanced chest CT) is rapidly performed [11,12]. Reserving consecutive time periods on a designated scanner for suspected or confirmed covid-19 cases is another practical option to manage workflow and reduce cleansing's ensuing down-time [7,12]. Alone in the CT room, the radiographer performs the exam. Images are then transferred to the radiologist for interpretation [9,12]. Each contact with a high-risk patient is to be followed by CT gantry and all possibly contaminated surfaces disinfection using vendor's suggested detergent solutions [7].

## 3 Chest computed tomography

### 3.1 Chest CT’s role in diagnosis and management

Confirmatory diagnosis of Covid-19 infection relies on the real-time reverse transcription-polymerase chain reaction (RT-PCR) nucleotides detection from respiratory tract samples [13]; a method with several shortcomings: High specificity but limited sensitivity (60–70%) generating false negative results [14,15], especially at early disease phases [16]. These false negatives may be due to an insufficient viral load in specimens-the lower respiratory tract samples being the most sensitive [17]-, inadequate extraction techniques, sampling timing [18], differences in RT-PCR tests sensitivity and laboratory errors [15,[19], [20], [21]]. They require reiterated assays that burden potentially insufficient infrastructure, overload the testing kits supply and impede quarantine measures with the possibility of unchecked contaminations [22,23]. Biological samples analysis is time-consuming [9]. The consequent delay in results obtainment obstructs the timely needed decisions of maintained isolated medical surveillance or discharge of suspected patients under investigation [11,18]. Chest CT complements viral nucleic acid detection with a considerable sensitivity reaching 97% in epidemic territories [14]. Rapid, simple to carry out and available, it may show typical Covid-19 imaging features preceding RT-PCR tests positivity in the initial disease stages [24]. In this setting, isolation measures are undertaken as quickly as possible to prevent additional infection spread [19]. Moreover, CT examination shows both disease evolution and gravity while RT-PCR only makes a positive diagnosis [19].

### 3.2 COVID-19 pneumonia's chest CT features

#### 3.2.1 Imaging findings

Initial Covid-19 CT features comprise bilateral, peripheral or posterior, multilobar ground-glass opacities, predominantly in the lower lobes and less commonly within the middle lobe. Consolidations superimposed on ground-glass opacifications are found in few cases, preponderantly in the elderly. In later disease stages, GGO transformation into multifocal consolidations, thickened interlobular and intralobular lines, crazy paving, traction bronchiectasis, pleural thickening, and subpleural bands are reported. Pleural/pericardial effusion, enlarged mediastinal lymphadenopathies, cavities, CT halo sign, and pneumothorax are infrequent but can be observed with pneumonia's evolution [4].

**Fig. 1:**
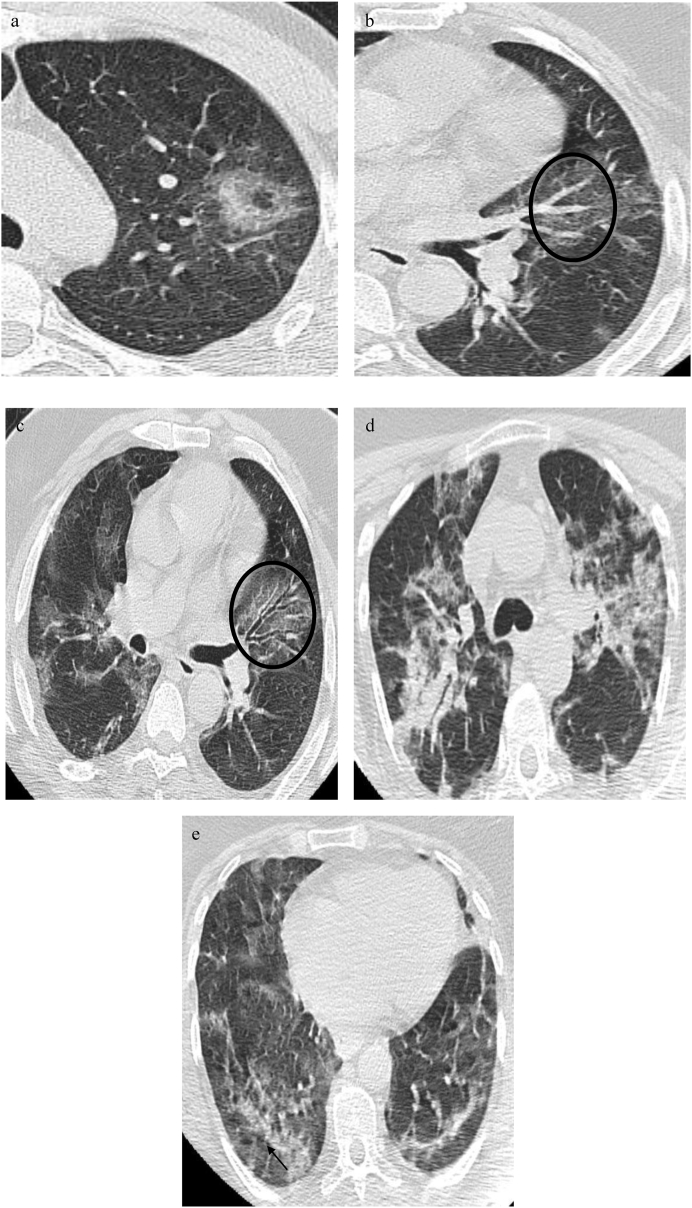
Covid-19 imaging features in patients with a positive RT-PCR at different disease stages. Unenhanced axial CT images of the lung show.***(a) A left upper lobe unifocal rounded ground glass opacity;******(b) Patchy peripheral ground glass opacity with vascular dilatation (Black circle);******(c) Multifocal, bilateral subpleural ground glass opacities with traction bronchiectasis (Black circle);******(d) Extensive bilateral ground glass opacities associated with thickened interlobular and intralobular septa (Crazy paving) alongside with peripheral consolidations;******(e) Subpleural band in advanced-phase disease (Black arrow).***

Dai et al. evaluated 234 covid-19 patients' high-resolution chest CTs. 15 (6,4%) exams showed no abnormalities. Abnormal attenuations in several bilateral lung lobes were described in 192 cases (87.67% 192/219). Anomalies concerned the entire lung in 121 patients (63.02%, 121/192), and only 16 patients (7.3%, 16/219) presented an isolated lobe lesion. Lesions were located in the lower lungs and/or lung periphery in 208 cases (94.98%, 208/219), and appeared in an irregular (88.3%, 193/219), little patches (86.3%, 189/219), bands-like (69.41%, 152/219), circular (49.32%, 108/219) and ‘anti-butterfly’ fashion (47.95%, 105/219). 60 patients (27.4%, 60/219) had an underlying pulmonary disease; emphysema most frequently (88.33%, 53/60), succeeded by bronchiectasia (16.67%, 10/60). Other reported signs included in order of frequency: vascular enhancement sign, interlobular septal thickening, air bronchogram, bronchiectasis, pleural thickening, solid nodules, reticular sign, fissures shift and bronchial walls thickening. 29 patients presented slight pleural and pericardial effusions. In 21 cases, mediastinal lymphadenopathy was depicted [21] (Fig. 1).

Li et al. retrospectively analyzed chest CT images of 131 confirmed Covid-19 patients from 3 chinese hospital establishments. 6 initial CT scans were normal whilst the remaining 125 examinations revealed bilateral lung participation in 104 cases (79%). In 100 cases (76%); lesions' distribution was peripheral.115 patients (87%) showed multiple abnormalities (>3). 106 patients (81%) presented patchy ground glass opacities, 91 patients (69%) had patchy consolidations and 40 patients (31%) exhibited nodules. 10 patients displayed diffuse, bilateral pulmonary involvement with ‘white lungs’ appearance. Interlobular septal thickening was described in 68 cases (52%) and crazy paving in 8 cases. Vascular enhancement sign, air bronchograms and fibrosis signs were also reported. Unusual findings were: isolated nodules in 7 cases only, reversed halo sign, pleural thickening, pleural/pericardial effusions and mediastinal lymphadenopathy. 1 case of consolidation with a cavity was reported, and represented an exceptional finding [25].

In Guan et al.’ study, two chest radiologists individually examined fifty-three thin-section chest CTs of confirmed COVID-19 patients. 47 patients (88.7%) presented Covid-19 pneumonia findings whilst 6 scans were normal (11.3%). Total cases showed ground glass opacities, among which 59.6% were circular and 40.4% patchy. 89.4% of cases presented crazy-paving, 63.8% consolidations, 57.5% stripes and 76.6% air bronchograms. Two cases presented pulmonary nodules. No cavities, pleural effusions nor enlarged mediastinal lymphadenopathies were detected. Bilateral pulmonary involvement took place in 78.7% of cases and concerned mainly the lower lobes (left lower lobe 85.1%, right lower lobe 72.3%). Peripheral subpleural distribution was described in 93.6% of patients; among them 25.5% displayed associated peri-bronchovascular repartition; 4.3% manifested a prevailing peri-bronchovascular localization and one severe case manifested diffuse spread [26].

In a single-center retrospective study including 99 real-time RT-PCR confirmed cases of 2019-nCoV in Wuhan Jinyintan Hospital, Chen et al. investigated radiological aspects. Bilateral pneumonia was reported in 74 cases (75%) and pneumothorax occurred in one case (1%) [27].

Gao et al. retrospectively examined high-resolution chest CTs of 6 patients diagnosed with Covid-19. The observed radiological characteristics aligned with those previously reported except one patient who exhibited tree-in-bud sign; an exceptional finding in viral infections that can be viewed in cases of immune disorders [28,29].

Yuan et al. Long et al. and Chung et al. retrospectively studied 27, 36 and 21 chest CTs of patients with confirmed Covid-19 pneumonia, respectively [6,30,31]. Consistently with the aforementioned novel coronavirus (2019-nCoV) infected pneumonia CT features, their findings aligned and included: multiple preponderant ground glass opacities combined with consolidations, predominantly peripheral or in a mixed central and peripheral distribution, bilateral, mainly involving lower lung zones; the right lower lobe being the most frequently concerned [31]; a location possibly favored owing to the right bronchus straight and short anatomical structure. Rarely encountered signs comprised: pure consolidation, sole central distribution, tree-in-bud, cavitation, mediastinal lymphadenopathy and pleural effusion [6,30,31].

#### 3.2.2 Evolution patterns

Based on the disease phase when scanning is performed, Covid-19 pneumonia's imaging features differ (Fig. 1).

Jin et al. characterized CT findings of five Covid-19 temporal stages. The ultra-early stage; in which asymptomatic patients mainly present sole or several focal ground glass opacities, patchy consolidations, nodules surrounded by ground glass attenuations and air bronchograms. The early stage (1–3 days after clinical manifestations) where CT exhibits one or numerous ground glass opacities associated with thickened interlobular septa. The rapid progression stage CTs (3–7 days following clinical symptoms) demonstrate sizeable pulmonary consolidative opacities with air bronchograms. The consolidation stage CTs (7–14 days beyond clinical symptoms onset) may show consolidations’ extent and density lessening. The dissipation stage (2–3 weeks following onset) is marked by the presence of fewer spotted consolidative opacities, band-like opacities as well as thickened bronchial walls and interlobular septa [32].

In a study including 63 confirmed Covid-19 patients, Pan et al. examined follow-up chest CTs performed 3–14 days after initial examinations. Identified disease progression imaging features included ground glass opacities and consolidations’ increase in extent, nodules increase in number, enlargement, confluence and for some, density reduction. Fibrous stripes appearance was associated with recovery whilst a “white lungs” appearance indicated worsening [33].

Pan et al. assessed covid-19's imaging features temporal course in a study including 21 confirmed patients. 4 stages were determined from clinical manifestations onset. In early stage (0–4 days), 4 patients had normal CTs and developed anomalies in the subsequent studies. Uni or bilateral, lower lobes, subpleural ground glass opacities were the major finding. Progressive stage (5–8 days) features consisted of two-sided, multilobar, extensive ground glass attenuations, consolidative opacities and crazy-paving. In peak stage (9–13 days), lesions' extent increased to a peak and dense consolidations prevailed. In absorption stage (14 days), as consolidative opacities and crazy-paving resolved; diffuse ground glass attenuations appeared [34].

Shi et al. grouped 81 Covid-19 patients based on the span separating clinical manifestations onset and the first chest CT. Group 1 (subclinical patients) presented prevailing one-sided multifocal ground glass attenuations. Group 2 (≤1 week following symptoms) showed two-sided extensive abnormalities transitioning from ground glass to consolidative and mixed opacities. Mediastinal lymph nodes enlargement and pleural effusion were noted. Group 3 (>1 week-≤2weeks) exhibited consolidative opacities alongside with interstitial alterations (bronchiectasis and thickened interlobular septa) suggestive of fibrosis. Group 4 (>2weeks to 3 weeks) findings consisted of predominant consolidative and mixed opacities, pleural thickening/effusion and bronchiolectasis.

Serial CTs demonstrate lung lesions’ worsening or amelioration; which helps predict outcome.

The commonest evolution pattern across consecutive CT scans was evolvement to a peak point succeeded by radiographic amelioration [35].

**Fig. 2:**
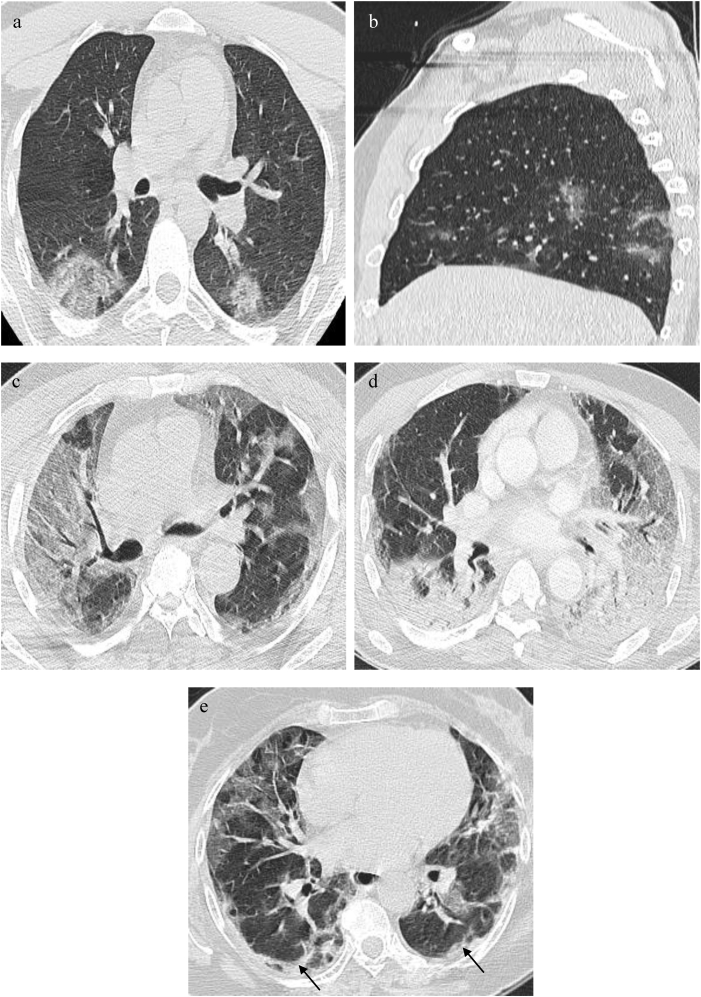
Covid-19 pneumonia's chest CT imaging features' changes over time.***(a,b) Early stage: Ground glass opacities involving the lower lobes with partial crazy paving;******(c) Progressive stage: Ground glass opacities extension and increased crazy paving;******(d,e) Peak stage: Consolidative opacities, sub-pleural lines (Black arrow) and bronchiectasis.***

A prospective study analyzing 41 Covid-19 patients data reported a median time from symptoms onset to both intensive care unit admission and assisted artificial ventilation of 10.5 days (IQR 8.0–17.0) and 10.5 days (IQR 7.0–14.0), respectively [36] (Fig. 2).

#### 3.2.3 Correlation with histopathological anomalies

The small sized virions deposit on the peripheral lung lobules leading to alveolar epithelium sloughing and alveolar wall break-down thus substituting the alveoli by exsudates, hyaline membrane, epithelium and cell debris. Interstitium hyperplasia, bronchioles edema, vascular congestion and microthrombi occur as well; all impacting several adjoining lobules [[37], [38], [39], [40], [41]].

In SARS’ acute phase (<11 days), disseminated alveolar damage is noted. In the delayed phase, disseminated alveolar damage, fibrous and organized pneumonia might be seen [42].

On the grounds of pathological alterations of SARS-Cov pulmonary infection; ground glass opacities may be the result of fractional airspaces filling. Consolidation may be consequent to disseminated alveolar damage, whilst bands spring from interstitial thickening or fibrosis [42].

#### 3.2.4 Severity

Critically ill patients' chest CTs demonstrate two-sided subsegmental and multi-lobar consolidations [4]. Over the course of a few days, rapid progression is noted and scans may exhibit ‘white lungs’ appearance [1].

**Fig. 3:**
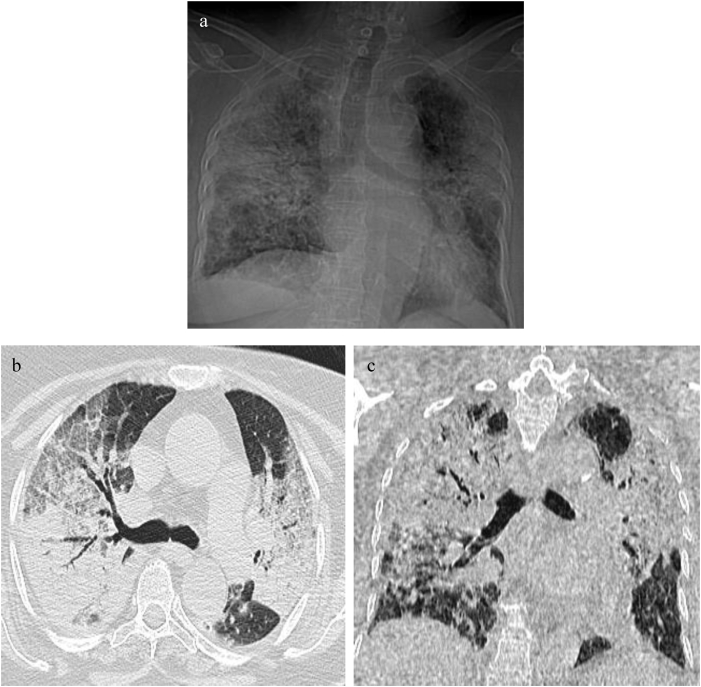
A 40 year-old-patient with a history of ten days of fever and coughing presented to the emergency department with an 86% O2 saturation and a 32/min respiratory rate. Frontal chest radiography (a) revealed extensive multilobar consolidations. Chest CT axial (b) and coronal (c) images showed typical severe peak-stage Covid-19 pneumonia imaging features with extensive ground glass opacities, crazy paving pattern, multilobar consolidations and bronchiectasis.

Yuan et al. investigated radiologic features association with mortality in a retrospective study including 27 patients with confirmed novel coronavirus 2019 infected pneumonia. In the mortality group; extensive and rapidly progressive multilobar consolidative opacities were present; indicating grave clinical evolution [30]. Pathologically, they correspond to diffuse alveolar injury, a recognized poor prognosis marker in H1N1, H5N1, H7N9 and SARS pneumonias [30,43,44] (Fig. 3).

#### 3.2.5 Complications

**Fig. 4:**
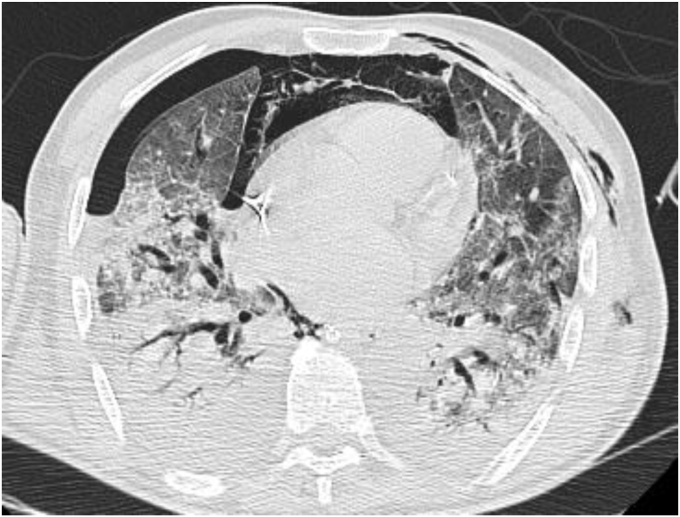
Axial chest CT image in a patient with severe advanced stage Covid-19 pneumonia complicated by right pneumothorax, pneumomediastinum and sub-cutaneous emphysema under mechanical ventilation.

Supplemental oxygen requirements raise suspicion of complications and indicate repeat CTs. CT identifies signs of Covid-19 myocardial injury induced pulmonary edema, superinfection, pulmonary thromboembolism when contrast-enhanced, progression towards acute respiratory distress syndrome and pneumothorax under mechanical ventilation … [45] (Fig. 4).

#### 3.2.6 Systematic reviews and meta-analysis reporting COVID-19 pneumonia's imaging features

Covid-19 pneumonia's imaging features have been extensively investigated. Among 72 systematic reviews and meta-analyses retrieved from pubmed, 20 were summarized in Table 1, based on the following criteria: recent articles published in english, and including patients with confirmed Covid-19 infection, large sample size, sufficient imaging data, and rigorous study design. High statistical heterogeneity was noted, due probably to differences in patient populations and study settings.

**Table 1:** Summary of systematic reviews and meta-analysis key findings.

Author/Year/Journal	Article type	Imaging modality	Key findings	Number of studies	Number of patients	Age (y-Old)	Effect estimate [95% CI]	P value	Heterogeneity (I^2^)%	P value	P value of publication bias tests
Zarifian et al [46]. February 2020 Clinical imaging .	Systematic review and meta-analysis	Chest CT	*Imaging findings*	103	9907						
			GGO±consolidation	66	6224		0.771(0.722-0.814)		89.5		0.001
			Reticulation±GGO	41	2667		0.462(0.385-0.541)		90.21		0.652
			Consolidation	44	4397		0.355(0.288-0.429)		96.06		0.825
			Organizing pneumonia	33	2557		0.368(0.289-0.455)		92.25		0.912
			Pleural effusion	48	3963		0.069(0.050-0.094)		78.25		0.001
			Lymphadenopathy	39	3197		0.051(0.035-0.075)		88.05		0.001
			*Distribution*								
			Bilateral distribution	70	5505		0.757(0.707-0.800)		89.78		0.602
			Central	26	2160		0.061(0.038-0.094)		91.73		0.001
			Diffuse	28	2080		0.351(0.267-0.444)		89.54		0.069
			Peripheral	43	3216		0.656(0.582-0.723)		94.29		0.002
			Middle lobe	15	1487		0.478(0.354-0.606)		94.44		0.004
Rodriguez-Morales et al [3]. March 2020 Travel Medicine and Infectious Disease	Systematic review and meta-analysis	Chest x-ray	*Pneumonia compromise*	19	2874	51.97 (46.06–57.89)					Egger’s test P=0.801
			-Unilateral pneumonia	7	316		0.25(0.052-0.448)	<0.001	96.37		
			-Bilateral pneumonia	9	557		0.729 (0.586-0.871)	<0.001	98.28		
			*Imaging findings*								
			-Ground glass opacities	10	584		0.68(0.518-0.852)	<0.001	99.09		
Borges do Nascimento et al [2]. April 2020 Journal of clinical medicine	Scoping review and meta-analysis	Chest x-ray Chest CT	Uni or bilateral chest opacities ± pleural effusion	61	59 254 22	3 months-99 years					
			Multiple GGO		20						
			Infiltrate		4						
			Normal findings		6						
			GGO (± septal thickening)		1204						
			Infiltration abnormalities		9						
			Parenchymal consolidation		325						
			Normal CT		8						
Sun et al [47]. May 2020 Quantitative Imaging in Medicine and Surgery	Systematic review and meta-analysis	Chest x-ray and CT	*Lesions’ distribution*	55	6616	48 (45.1–50.9)					
			Bilateral involvement				0.780(0.450-1)			<0.001	
			Unilateral involvement				0.203(0.099-0.300)			<0.001	
			Peripheral distribution				0.653(0.259-1)			<0.001	
			Central distribution				0.035(0.009-0.098)			<0.001	
			Peripheral & central distribution				0.311(0.019-0.740)			0.102	
			*Imaging findings*								
			Normal imaging				0.133(0.007-0.384)			<0.001	
			GGO				0.580(0.166-1)			<0.001	
			Consolidation				0.441(0.016-0.714)			<0.001	
			GGO & consolidation				0.529(0.190-0.767)			<0.001	
			Interlobular septal thickening				0.229(0.009-0.804)			<0.001	
			Crazy-paving pattern				0.235(0.031-0.916)			<0.001	
			Pleural effusion				0.110(0.009-0.804)			<0.001	
			Air bronchogram				0.425(0.077-0.803)			<0.001	
			Lymphadenopathy				0.048(0.009-0.084)			0.084	
			Nodules				0.116(0.020-0.428)			<0.001	
			Linear opacity				0.412(0.074-0.650)			<0.001	
Chang et al [48]. May 2020 Journal of the Formosan Medical Association	Systematic review and meta-analysis	Chest CT	Patchy consolidations	3	93		0.31(0.13-0.55)		51	0.09	
			Ground glass opacities				0.48(0.36-0.64)		0	0.52	
			No lesion				0.27(0.18-0.43)		0	0.64	
Elshafeey et al [49]. July 2020 International journal of gynecology and obstetrics	Systematic scoping review	Chest CT	*Lesion’s distribution*	33	385	21-42					
			Bilateral pneumonia		99/125(79.2%)						
			Unilateral pneumonia		22/125(17.6%)						
			*Imaging findings*								
			No lesions		4/125(3.2%)						
			GGO		102/125(81.6%)						
			Consolidation		22/125(17.6%)						
			Reticular		1/125(0.8%)						
			Pleural thickening		1/125(0.8%)						
			Pleural effusion		9/125(7.2%)						
			Atelectasis		1/125(0.8%)						
			Crazy-paving		1/125(0.8%)						
Salehi et al [4]. July 2020 American Journal of Roentgenology	Systematic review	Chest CT	*Lesions’ distribution*	30	919						
			Bilateral involvement	12	435/497(87.5%)						
			Peripheral distribution	12	92/121(76%)						
			Posterior involvement	1	41/51(80.4%)						
			Multilobar involvement	5	108/137(78.8%)						
			*Imaging findings*								
			GGO	22	346/393(88%)						
			Consolidation	10	65/204(31.8%)						
Kumar et al [50]. July 2020 Journal of tropical pediatrics	Systematic review and meta-analysis	Chest CT Chest x-ray Chest US	*Imaging findings*	46	923	<19					Considered high (all the included studies were either case series or case reports)
			GGO	32	706		0.39(0.31-0.48)		82	0.00	
			Consolidation	12	192		0.23(0.12-0.34)		82	0.00	
			Halo sign	6	78		0.26(0.11-0.41)		51	0.09	
			Patchy shadow	13	246		0.44(0.32-0.55)		62	0.00	
			Prominent bronchovascular markings	5	97		0.17(0.09-0.24)		0.0	0.83	
			Bronchial wall thickening	4	36		0.11(0.01-0.21)		0.0	1.00	
			Pleural effusion	5	187		0.02(0.001-0.04)		0.0	0.60	
			Interstitial pattern	4	187		0.12(0.01-0.23)		82	0.00	
			Nodules	5	63		0.25(0.09-0.41)		62	0.03	
Cao et al [1]. September 2020 Journal of medical virology	Systematic review and meta-analysis	Chest CT	*Pneumonia compromise*	31	46 95	46.62 (31.71-61.53)					Egger’s test P =0.091
			Unilateral pneumonia		522		0.201(0.106-0.302)	<0.001	97.605	<0.001	
			Bilateral pneumonia		1196		0.755(0.639-0.871)	<0.001	98.736	<0.001	
			*Imaging findings*								
			Lung consolidation		122		0.369(0.215-0.523)	<0.001	91.717	<0.001	
			Ground-glass		1413		0.699(0.602-0.796)	<0.001	98.651	<0.001	
			Air bronchogram		119		0.513(0.326-0.701)	<0.001	89.834	<0.001	
			Grid-form shadow		64		0.244(0.116-0.371)	<0.001	87.365	<0.001	
			Bronchovascular bundles thickening		41		0.395(0.082-0.708)	0.013	95.592	<0.001	
			Hydrothorax		23		0.185(0.001-0.370)	0.049	97.871	<0.001	
			Irregular or halo sign		107		0.544(0.255-0.833)	<0.001	96.217	<0.001	
Cui et al [51]. September 2020 Journal of medical virology	Review	Chest CT	*Imaging findings*	24	2597	< 1 year: 446 (17.9%) 1 -5 years: 593 (23.8%) 6 - 10 years: 626 (25.1%) 11 - 15 years: 492 (19.7%) >15 years: 335 (13.4%)					
			Normal imaging		178/409(43.5%)						
			Ground-glass opacity		87/294(29.6%)						
			Local patchy shadowing		60/294(20.4%)						
			Bilateral patchy shadowing		43/294(14.6%)						
			White lung change		2/409(0.5%)						
			Pleural effusion		3/409(0.7%)						
Wan et al [52]. July 2020 Academic radiology	Systematic review and meta-analysis	Chest CT	*Imaging findings*	14	1115	48.02					
			GGO	13	1073		0.690(0.580-0.800)		96.7		
			Consolidation	14	1115		0.470(0.350-0.600)		95.6		
			Air bronchogram	8	565		0.460(0.250-0.660)		97.6		
			Crazy-paving	7	695		0.150(0.080-0.220)		89.1		
			*Extent*								
			RLL	8	547		0.700(0.460-0.950)		98.6		
			≥3 lobes	7	438		0.650(0.580-0.730)		63		
			All 5 lobes involved	8	489		0.420(0.320-0.530)		84.4		
			*Distribution*								
			Peripheral	9	817		0.670(0.550-0.780)		93.6		
Awulachew et al [53]. July 2020 Radiology Research and Practice	Systematic review and meta-analysis	Chest CT	*Imaging finding*	60	5041	49±11.6					0.1947
			GGO with consolidation		768 (18%)						
			GGO		2482(65%)						
			Consolidation		1259(22%)						
			Crazy-paving		575(12%)						
			Reversed halo sign		146(1%)						
			Interlobular septal thickening		691(27%)						
			Air bronchogram sign		531(18%)						
			*Distribution*								
			Bilateral		3952(80%)						
			Unilateral		641(20%)						
			Right lung		48(62%)						
			Left lung		29(38%)						
			LUL		731(74%)						
			LLL		504(46%)						
			RUL		455(40%)						
			RML		326(38%)						
			RLL		784(74%)						
			*Extent*								
			One lobe		278(14%)						
			Two lobes		299(11%)						
			Three lobes		250(13%)						
			4 lobes		212(15%)						
			5 lobes		384(34%)						
			>one lobe		1145(76%)						
			*Other findings*								
			Pleural effusion		94(1.6%)						
			Lymphadenopathy		21(0.7%)						
			Pulmonary nodules		262(9%)						
			*Follow-up findings*								
			Early disease : Pure GGO followed by Crazy-paving								
			Later disease course : Consolidation, prominent bilateral involvements								
Jutzeler et al [54]. August 2020 Travel Medicine and Infectious Disease	Systematic review and meta-analysis	Chest CT	*Imaging findings*	148	12′149	47(35-64.4)					Egger’s test: p < 0.05
			GGO	62	2446/5591		0.691(0.568-0.792)		97.9		
			Consolidation	30	771/2022		0.383(0.269-0.511)		92.1		
			GGO with consolidation	15	153/323		0.495(0.405-0.587)		43.1		
			Nodular lesions	13	70/1345		0.153(0.073-0.295)		83.3		
			Reticulation/interlobular septal thickening	7	81/1244		0.218(0.051-0.593)		95.8		
			Crazy-paving pattern	5	59/210		0.307(0.138-0.550)		75.2		
			Pleural effusion	10	2745/4247		0.078(0.050-0.121)		94.6		
			*Lesions’ distribution*								
			Bilateral pneumonia	48	52/666		0.772(0.708-0.831)		55.6		
			Unilateral pneumonia	32	799/3745		0.192(0.164-0.224)		73		
Mouhand F. et al [55]. August 2020 The American journal of tropical medicine and hygiene	Systematic review and meta-analysis	Lung US	*Imaging findings*	7	122	>18years					
			B-pattern	7			0.97 (0.94–1.00)		0		
			Pleural line abnormalities	5			0.70 (0.13–1.00)		96		
			Pleural thickening	5			0.54 (0.11–0.95)		93		
			Subpleural or pulmonary consolidation	6			0.39 (0.21–0.58)		72		
			Pleural effusion	5			0.14 (0.00–0.37)		93		
Syed Zaki et al [56]. September 2020 Le Infezioni in Medicina	Systematic review and meta-analysis	Chest CT	*Distribution of lesion*	54	2693						
			-Bilateral		1411/1937		0.741(0.684-0.795)		85.76		
			-Subpleural		299/509		0.572(0.390-0.743)		93.08		
			-Peripheral		770/1287		0.571(0.467-0.671)		92.42		
			-Posterior		44/69		0.379(0.015-0.872)		92.38		
			-Unilateral		216/1048		0.205(0.150-0.266)		77.48		
			-Central		47/551		0.089(0.031-0.172)		87.84		
			*Lobe involvement*								
			-LLL		500/696		0.715(0.589-0.821)		90.91		
			-RLL		504/710		0.665(0.534-0.785)		91.45		
			-LUL		403/681		0.572(0.444-0.694)		90.39		
			-RUL		385/690		0.531(0.418-0.642)		87.66		
			-RML		345/736		0.428(0.291-0.571)		93.24		
			*Imaging findings*								
			GGO		1541/2416		0.646(0.576-0.714)		91.52		
			Mixed		524/1176		0.430(0.368-0.493)		72.56		
			Consolidation		615/2037		0.277(0.191-0.371)		95.19		
			Septal thickening		399/846		0.406(0.282-0.537)		92.74		
			Air bronchogram		491/1199		0.397(0.291-0.509)		93.26		
			Fibrosis/stripes		200/562		0.372(0.217-.0.541)		93.98		
			Crazy-paving		350/1181		0.290(0.190-0.401)		93.68		
			Subpleural lines		117/698		0.150(0.072-0.251)		90.81		
			Nodules		95/918		0.112(0.065-0.170)		83.28		
			Pleural effusion		108/1784		0.058(0.041-0.077)		62.31		
			Lymphadenopathy		65/1193		0.053(0.029-0.084)		72.11		
			Pericardial effusion		13/394		0.030(0.013-0.054)		33.16		
Xu et al [57]. October 2020 European radiology	Systematic review and meta-analysis	Chest CT		16	3186	37-62	0.92(0.86-0.96)		96.4		The risks of bias in all studies were moderate.
			Sensitivity	14	2689		0.25(0.22-0.3)				
			Specificity	2	419		0.33(0.23-.44)				
Choi et al [58]. November 2020 European journal of radiology	Systematic review and meta-analysis	Brain CT and MRI	Cerebral microhemorrhages	21	2125	>58 years	0.069(0.049-0.089)	<0.001	94		<0.001
			Spontaneous acute ICH				0.054(0.031-0.076)	<0.001	87		<0.001
			Acute/subacute infarct				0.240(0.160-0.318)	<0.001	97		0.014
			Encephalitis/encephalo-pathy				0.330(0.019-0.047)	<0.001	92		<0.001
Garg et al [59]. November 2020 Clinical Imaging	Systematic review and meta-analysis	Chest x-ray Chest CT	*Imaging findings*	56 5	6007 396	2.1-70					
			GGO				0.387(0.222-0.583)		83		
			Consolidation		5762		0.469-0.297-0.649)		84		
			*Imaging findings*								
			GGO				0.669(0.608-0.724)		92		
			GGO+Consolidations				0.449(0.387-0.513)		83		
			Consolidation				0.321(0.236-0.419)		96		
			Crazy-paving				0.291(0.196-0.408)		93		
			Halo sign				0.236(0.117-0.418)		94		
			Nodule				0.089(0.057-0.138)		65		
			Pleural effusion				0.056(0.042-0.074)		51		
			Lymphadenopathy				0.027(0.013-0.055)		84		
			*Diagnostic accuracy estimates*								
			GGO sensitivity/specificity				0.73(0.71-0.80)/0.61(0.41-0.78)		96/94		
			GGO+Consolidation sensitivity/specificity				0.58(0.48-0.68)/0.58(0.41-0.73)		31/77		
			Consolidation only sensitivity/specificity				0.49(0.20-0.78)/0.56(0.30-0.78)		96/91		
Islam et al [60]. December 2020 Frontiers in medicine.	Review	Chest CT	Early identification Reduces transmission especially in asymptomatic patients	13	235	25-40					
			*Imaging findings*								
			GGO								
			Patch-like shadows								
			Fiber shadows								
			Pleural effusion								
			Pleural thickening								
			*Distribution*								
			Bilateral, multi-lobe distribution Peripheral, random, and diffuse involvement								
Nino et al [61]. January 2021 Pediatric Pulmonology	Systematic review and meta-analysis	Chest CT	*Imaging findings*	29	1026	6.57 (1.5–14.5)					
			Normal imaging				0.375(0.275-0.440)	<0.001	86.31		
			GGO				0.372(0.293-0.450)	<0.001	85.76		
			Consolidations/pneumonic infiltrates				0.223(0.178-0.269)	<0.001	94.54		
			*Distribution*								
			Bilateral compromise				0.277(0.199-0.356)	<0.001	87.59		

In a study involving 919 patients, Salehi et al. report the following initial characteristic CT aspects: multilobar, bilateral, principally in the lower lobes, peripherally distributed ground glass opacification. Later, in the intermediate course of the disease, ground glass opacities increase in size and number gradually turning into multifocal consolidations. Septal thickening and crazy-paving develop [4]. Mixed ground glass opacities and consolidations are frequently found. Septal thickening, bronchiectasis and pleural thickening are less common. Pleural effusion, pericardial effusion, mediastinal lymphadenopathy, cavities, halo sign and pneumothorax are rare however possible; detected with disease progression. In certain studies; Covid-19 CT features differed in relation to age with older patients presenting more atypical findings and consolidative opacities than younger ones in which ground glass attenuations prevailed [4,62,63].

In a systematic review and meta-analysis including 46 959 patients, Cao et al. reported the following two main chest CT aspects: bilateral lung involvement (75.7%, 0.639–0.871) and ground glass opacities (69.9%, 0.602–0.796), followed by halo sign (54.4%, 0.255–0.833), air bronchograms (51.3%, 0.326–0.701), thickening of bronchovascular bundles (39.5%, 0.082–0.708), grid-like shadows (24.4%, 0.116–0.371) and hydrothorax (18.5%, 0.001–0.370). Nodules, stripes, vascular enhancement sign, bronchial wall thickening, anti-halo and mosaic signs … were also described [1].

In a scoping review and meta-analysis of 59 254 patients imaging data, Borges do Nascimento et al. stated that the most frequently encountered abnormalities amongst patients who undertook chest radiography were bilateral opacities, multifocal ground glass shadows, infiltrations and consolidations. 6 patients presented normal chest films.

Concerning computed tomography: 8 patients had normal CTs. Predominantly bilateral, peripheral, patchy ground glass opacities prevailed (associated or not with thickened septa), succeeded by consolidations. Ground glass nodules’ size increase and progression to alveolar consolidations were of note [2].

Rodriguez-Morales et al. reported Covid-19 pneumonia main chest x-rays features in a systematic review and meta-analysis comprising 2874 patients. Their findings consisted of bilateral pneumonia and ground glass opacities [3].

### 3.3 Diagnostic difficulties and differentials

In the presence of immune disorders or underlying pulmonary diseases (emphysema, fibrosis …), atypical Covid-19 imaging patterns can be observed and diminish diagnostic confidence [64]. Covid-19 temporal progression is another noteworthy element to take into account. Early (first two days after symptoms onset) negative CTs should not be relied on to exclude the presence of SARS-CoV 2 infection [33,65].

Although highly sensitive in diagnosing Covid-19 pneumonia, chest CT features in a screening population are non-specific [14]. Infectious (viral and bacterial pneumonia …) as well as non-infectious diseases (vasculitis, organizing pneumonia …) share Covid-19 imaging features complicating the differential diagnosis [66]. Viral pneumonia (Influenza A virus, Influenza B virus, SARS-CoV, MERS coronavirus cytomegalovirus, adenovirus, respiratory syncytial virus …) primarily exhibits interlobular septa, peri-bronchial and peri-vascular interstitial inflammation. On CT, numerous hilar and subpleural high attenuation reticulations are noted [67,68]. Bronchial wall inflammation clogs the bronchioles in part or in total and appears as focal edema or atelectasis on CT.

Covid-19 imaging features resemble those of SARS and MERS [69,70]. One could theorize that the SARS-CoV2 infected pulmonary parenchyma might react in a way comparable to the SARS and MERS lung recovery patterns [71]. Shared features are peripheral ground glass and consolidative opacities combined with crazy-paving. SARS usually presents as a unifocal one-sided lung opacity initially [72]. Progression is rapid. Consolidations affect multiple segments and lobes resulting possibly in ‘white lungs’ appearance [73]. Cavities and spontaneous pneumomediastinum are reported [74]. MERS primarily appears as extensive basilar and subpleural ground glass opacities with a scope greater than that of consolidations [75]. Pneumothorax and pleural effusion are common signs and indicate poor prognosis [76].

Bronchial and lobar pneumonia are bacterial pneumonias major manifestations. Their CT features comprise extensive irregular consolidations, mucoid impactions, bronchial walls thickening and centrilobular nodules. Pleural effusion and mediastinal lymphadenopathy are common [19].

Cryptococcus infections manifest as uni or multifocal subpleural nodules and consolidations [77].

Heart failure causes pulmonary alveolar and interstitial edema. Alveolar edema major CT findings are ground glass opacities and high-attenuations typically displaying the butterfly sign. Interstitial edema appears as bilateral interlobular septal thickening, peribronchial infiltration and blood-flow redistribution [78].

Studies assessing radiologists’ performance in accurately differentiating Covid-19 over other pneumonia on chest-CT are limited. In one investigation, Bai et al. evaluated radiologists accuracy in distinguishing Covid-19 from viral pneumonia; 424 abnormal chest computerized tomographies-among which 219 belonged to patients with RT-PCR confirmed SARS-Cov2 infection and 205 to patients with positive respiratory viral pneumonia-were selected and blindly examined by three chinese radiologists [66]. Their accuracies to differentiate Covid-19 from viral pneumonia were 83% (95 CI: 79–86%), 80% (95% CI: 76–83%), and 60% (95% CI: 55–65%), respectively [48]. In a comparable manner, accuracies of 4 U S radiologists in differentiating covid-19 from non covid-19 pneumonia in 58 age-matched scans chosen at random were 97% (95% CI: 88–100%), 88% (95 CI: 77–95%), 83% (95% CI: 71–91%), and 84% (95% CI: 73–93%), respectively. Specificity fluctuated from 93 to 100% and sensitivity from 70 to 93%. Authors concluded that chinese and U.S radiologists sensitivity to discriminate Covid-19 pneumonia from viral pneumonia on chest CT was moderate whilst specificity was high. Of note, the cohort size was small and non-infectious diseases with Covid-19 comparable features were not integrated [66].

### 3.4 Reporting templates

Current literature and expert consensus suggest standardized CT reporting templates use within the Covid-19 pneumonia context [79]. The goal is to guide radiologists, decrease reports inconsistencies and better clinicians' comprehension of radiologic features for a finer incorporation of imaging into decisions' adoption. The precise probability of Covid-19 pneumonia is not given. Four reporting categories are proposed based on the findings’ typicality of Covid-19 pneumonia aspects as indicated in the literature [79].

**Fig. 5:**
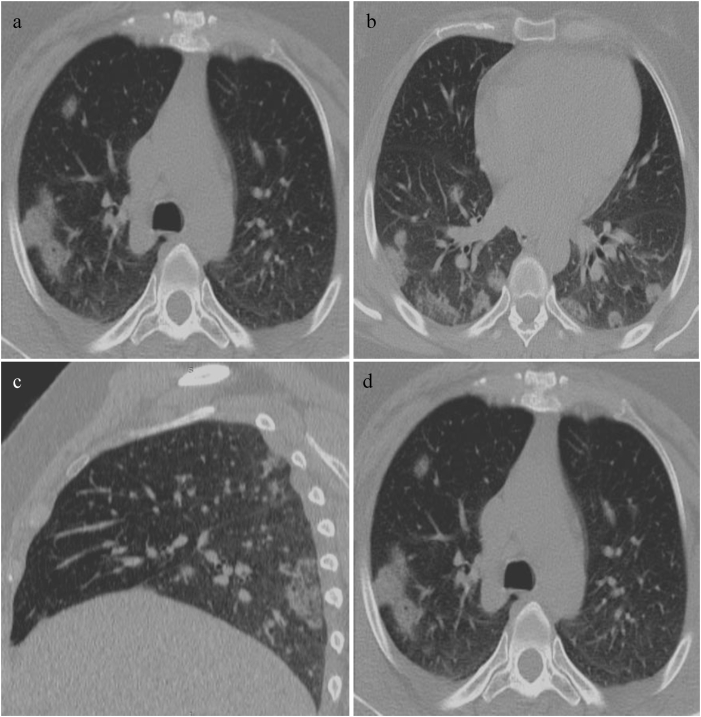
Typical Covid-19 imaging features in a 45-year-old woman with a positive RT-PCR. Unenhanced axial (a, b), sagittal (c) and coronal (d) CT images of the lung show multifocal, bilateral, posterior and peripheral rounded consolidations surrounded by ground glass opacities.

**Fig. 6:**
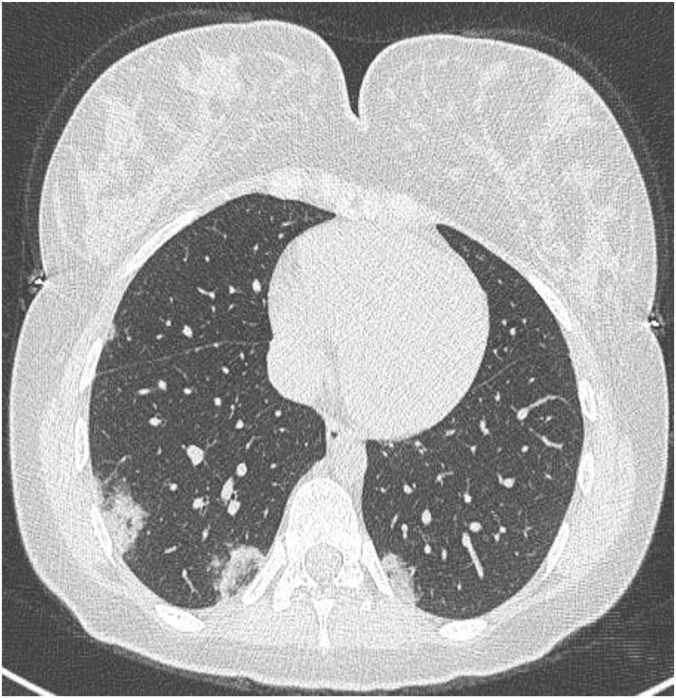
Axial CT image showing bilateral posterior, peripheral, and rounded ground glass opacities in a patient with organizing pneumonia secondary to dermatomyositis; typical Covid-19 imaging features.

“Typical appearance” findings are those commonly pointed out in the literature as most specific of Covid-19 pneumonia (bilateral, peripheral, multifocal, round ground glass attenuations associated or not with consolidative opacities, crazy-paving, reserve halo sign or other organized pneumonia features). The main differential diagnosis is Influenza pneumonia and organized pneumonia (connectivities, drug toxicity …) (Fig. 5, Fig. 6).

**Fig. 7:**
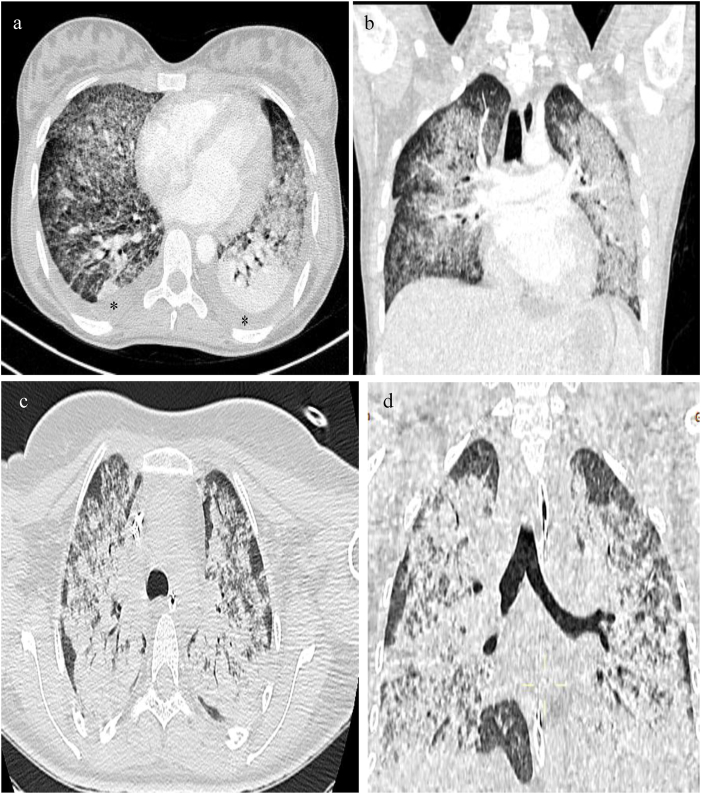
‘Indeterminate Covid-19 appearance’ Chest CT images in 2 patients showing ground glass opacities and consolidations with no specific distribution nor morphology in a case of.(a,b) Acute eosinophilic pneumonia associating interlobular septal thickening, lower lobes air space consolidations and pleural effusions (*).***(c,d) Extensive, multifocal, bilateral consolidation in a case of diffuse alveolar hemorrhage.***

“Indeterminate appearance” findings are nonspecific of Covid-19 pneumonia - extensive or small ground glass attenuations lacking a specific peripheral disposition and a circular shape -, hard to differentiate radiologically from many pathologies (alveolar hemorrhage, Pneumocystis pneumonia …) (Fig. 7).

**Fig. 8:**
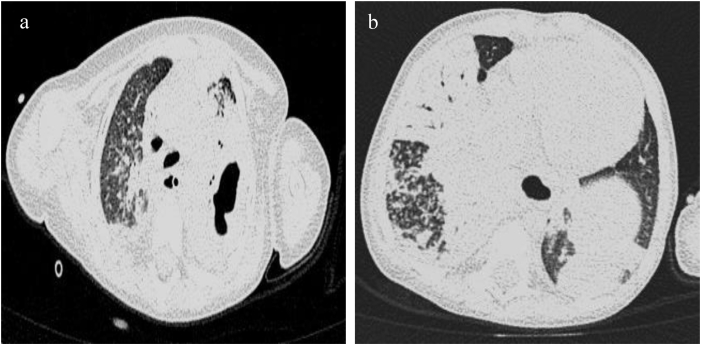
Atypical Covid-19 C T features. Unenhanced axial chest CT images showing segmental consolidations with no ground glass opacities, cavitation alongside with centrilobular and tree-in-bud nodules in an active tuberculous infection (a,b).

“Atypical appearance” findings are not or infrequently reported in the setting of Covid-19 pneumonia such as tree-in-bud or centrilobular nodules, sole lobar or segmental consolidative opacities, cavities … Bacterial pneumonia is one of the alternative diagnosis (Fig. 8).

“Negative for pneumonia” indicates the absence of infection related lung anomalies.

Of note, Covid-19 early stage CTs might be normal [79]. Describing accompanying underlying lung disease is important. Mixed typical and atypical imaging patterns coexistence (secondary infection, aspiration …), as well as incidental findings of Covid-19 pneumonia complicate grouping and mandate direct communication with the referring physicians [80].

## 4 Chest radiography

**Fig. 9:**
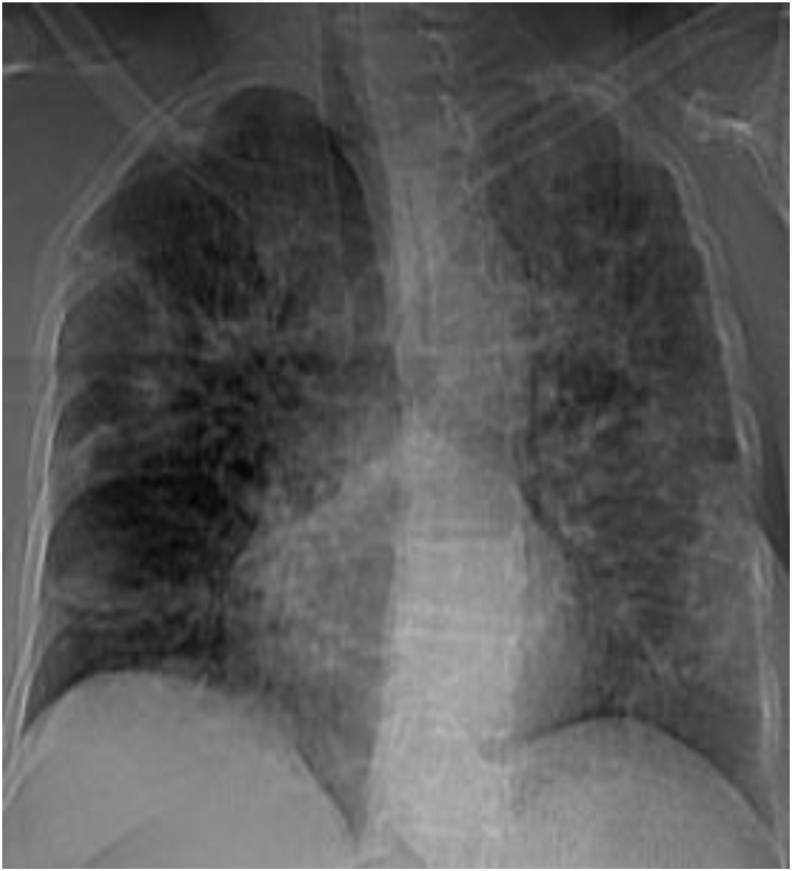
Frontal chest radiography showing bilateral air space consolidations in a Covid-19 patient.

Chest radiography's sensitivity for covid-19 pneumonia is limited for it fails to detect ground glass opacities; the infection's major manifestation [81]. In early disease phases, x-rays have little diagnostic input whilst CT features might precede symptoms onset [33,82]. In advanced disease, radiographs might exhibit progression towards acute respiratory distress syndrome [4]. Guan et al. reported a higher propensity of radiographic anomalies in severe disease (76.7% (46/60 patients with grave infection) versus 54.2% (116/214 cases with mild infection) [65] (Fig. 9). According to the British Society of Thoracic Imaging, when a chest radiography indicates an alternate diagnosis (lobar pneumonia, pneumothorax …) CT offers no extra value over clinical and laboratory evaluation [12]. Chest x-rays can be of use in hospitalized patients follow-up and complications assessment [83]. Standard day-to-day chest x-rays regiments for stable mechanically ventilated covid-19 patients are not indicated; as studies comparing daily to on-demand intensive care units patients imaging revealed no significant differences in mortality, length of stay nor mechanical ventilation days [8,84,85]. Material movability might favor radiograph's use in singled populations [8].

## 5 Lung ultrasound

Quick, available, inexpensive, fast to disinfect and non-invasive; ultrasound uses no ionizing radiations and is portable limiting highly contagious and possibly unstable SARS-Cov2 patients transfers, at the expense of operators' exposure [86]. Current data does not support lung ultrasound's use for Covid-19′ pneumonia diagnosis [87]. Although highly sensitive, ultrasonography exhibits no specific covid-19 features [87]. Reported findings comprise: pleural thickening, multifocal/coalescent B lines and consolidations; depending on the infection's phase and gravity [88]. A lines re-appearance is a recovery indicator [88]. Pleural effusion is unusual [88]. Its presence may prompt alternative diagnosis appraisal (heart failure, bacterial pneumonia …) [89]. Deep lesions with no extension to the pleural surface cannot be detected by lung ultrasonography [88]. Its utilization in intensive care units and at point of care allows distinction between various hypoxia causes (consolidation, interstitial syndrome, pulmonary edema, pleural effusion …), with a diagnostic accuracy superior to chest radiography permitting proper treatment's administration [[89], [90], [91]]. Other lung ultrasound applications include: Sars-Cov2 pneumonia severity and evolution assessment using the Lung Ultra-Sound Score potentially [92,93], directing mechanical ventilation techniques delivery (recruitment maneuvers, prone positioning, weaning …) and guiding extracorporeal membrane oxygenation therapy [88,93].

## 6 MRI

The American College of Radiology advocates minimizing MRI's utilization in suspected or confirmed SARS-CoV-2 patients [94]. A major limitation to MRI's use in COVID-19 pneumonia setting is imaging equipment disinfection challenges [95]. Due to cardiac and respiratory motion artifacts, pulmonary parenchyma low proton density, and air-soft-tissue interfaces induced susceptibility artifacts, pulmonary MRI indications have been traditionally limited [96]. Nonetheless, alveolar spaces pathology-namely GGOs and consolidations -appear hyperintense in comparison to environing tissues due to fluid accumulation and higher proton density [97]. In radiation at-risk groups such as children and pregnant females, chest MRI is a viable imaging alternative [96]. Yang et al. ultrashort echo time MRI (UTE-MRI) showed high concordance with CT in detecting Covid-19 pneumonia typical imaging features (GGOs, consolidations, GGOs with consolidation) [98]. In Ates et al.‘s study, MRI had 91.7% sensitivity, 100% specificity, 100% positive predictive value, 95.2% negative predictive value, and demonstrated no significant differences in detecting GGOs or consolidations compared to CT [97]. Multiple MRI sequences detected GGOs, consolidation, reticulation, and reverse halo sign in a case series of eight patients by Torkian et al. [99]. Among studied sequences, T2-weighted turbo spin-echo turbo inversion recovery magnitude (T2W TSE-TIRM) resolved lesions more brightly [99].

## 7 PET CT

While considerably sensitive, PET imaging's applicability as a first-line Covid-19 pneumonia diagnostic modality is limited due to its low specificity, high cost, radiation risk, lengthy staff exposure, and long scanner bays disinfection and suites' ventilation processes [100,101].

Early SARS-CoV-2 infection's detection in asymptomatic individuals, especially vulnerable populations (immunocompromised and oncology patients) allows timely supportive care initiation, which is critical to better outcome and improve survival. In patients requiring 18-F-FDG PET/CT for unrelated clinical indications (malignancy evaluation and staging), several reports demonstrated incidental hypermetabolic pulmonary foci in anatomic regions corresponding to Covid-19 related parenchymal abnormalities [101,102]. Jointly with PET imaging, a low-dose CT is performed, serves as a diagnostic tool, and might detect precocious pulmonary lesions justifying confirmative biological analyses [103]. In a case series of four patients who underwent 18 F-FDG PET/CT in the course of acute Covid-19 pneumonia, Qin et al. outlined parenchymal 18 F-FDG uptake in ground-glass and/or consolidative opacities regions with maximum standardized uptake (SUVmax) values varying from 4.6 to 12.2 [104]. Three patients presented nodal involvement. It was proposed that higher 18 F-FDG uptake could correlate with greater erythrocyte sedimentation rates and require more time to resolve [[104], [105], [106]]. In a systematic review including 52 patients, the mean SUV max of pulmonary lesions with18 F-fluorodeoxyglucose uptake was 4.9 ± 2.3 [107]. 18 F-FDG PET/CT could be of use in evaluating other organs’ alterations, particularly the heart, kidneys, and digestive tract [106]. Two patienst undergoing PET/CT for prostate cancer showed 68 Ga-labelled prostate-specific membrane antigen (68 Ga-PSMA) and 18 F-labelled choline (18 F-choline) uptake in subpleural GGO regions [108].

## 8 Artificial intelligence imaging applications

In the fight against Covid-19, artificial intelligence empowered imaging might strengthen available tools and help radiologists [109]*.* Scanning's automation relying on shifting CT tables and visual sensors enables contact-free imaging. Lung lobes and lesions segmentation is applied in Covid-19 diagnosis and quantification [109].

Artificial intelligence can assist both chest x-ray and CT Covid-19 screening, differential diagnosis and severity assessment.

In a study including x-rays of 70 covid-19 patients and 1008 non covid-19 pneumonias, Zhang et al. introduced a ResNet model to discern radiographic Covid-19 findings. Sensitivity and specificity were 96.0% and 70.7% with an AUC of 0.952 [110].

Similarly, Wang et al. applied a deep convolutional neural network founded model to identify Covid-19 radiographic features among chest x-rays images of 931 bacterial pneumonias, 45 Covid-19 pneumonias, 660 viral pneumonias and 1203 normal cases. The obtained testing accuracy was of 83.5% [111].

Chen et al. reported that with artificial intelligence performances aid; radiologists’ chest CT reading time is reduced by 65% [112].

To train and test a deep learning model for Covid-19 diagnosis; Zheng et al. used 540 chest CTs (313 covid-19 positives and 229 covid-19 negatives). Achieved sensitivity and specificity were 90.7% and 91.1% respectively, with an AUC of 0.959 [113].

In a similar manner, Jin et al. used a UNet and ResNet 50 combined model for abnormalities localization and diagnosis. Their study included 1136 chest CTs (723 with Covid-19 and 413 without covid-19 pneumonia). The sensitivity and specificity were 97.4% and 92.2% respectively [114].

To distinguish Covid-19 from typical viral pneumonia, Xu et al. testing dataset included chest CT images from 219 Covid-19 patients, 224 Influenza-A cases and 175 healthy subjects. Their model's overall accuracy was of 86.7% [115].

Likewise, Li et al. used a significant dataset comprising 4356 chest computed tomography images from 1296 Covid-19 pneumonias, 1735 community-acquired pneumonias and 1325 cases with no pneumonia. Their model's achieved sensitivity in depicting covid-19 was 90%, specificity 96% and AUC 0.96 [116].

To assess Covid-19 pneumonia's severity (severe or not), Tang et al. endorsed a deep learning method to segment the pulmonary parenchyma into anatomical regions [117]; on the basis of which, infection ratios were measured and employed as quantitative traits to instruct the model that analyzed chest CTs of 176 Covid-19 confirmed cases. A true positive rate of 93.3%, a true negative rate of 74.5% and an accuracy of 87.5% were noted [118].

In Covid-19 follow-up studies; artificial intelligence application is in its early phases and remains a pending question [109].

## 9 Imaging in pediatric patients

Cai et al. reported one-sided patchy infiltrates in 40% (4/10) of their covid-19 pediatric patients chest x-rays [119].

Xia et al. retrospectively analyzed chest CT features of 20 Covid-19 pediatric inpatients. Lung lesions presented a subpleural distribution. They were unilateral in 30% (6/20) and bilateral in 50% (10/20) of cases. 3 neonates and one infant had normal initial chest CTs (20%, 4/20). Ground glass attenuations were noted in 60% (12/20), Halo sign consolidation in 50% (10/20) and tiny nodules in 15% (3/20) of patients. No pleural effusion nor lymphadenopathy were reported [120].

In a review including 2597 Covid-19 pediatric patients, 409 children's chest CTs were available, among which 178 (43.5%) showed no anomalies, 2 (2/409, 0.5%) presented white lungs and 3 (3/409, 0,7%) pleural effusion [121]. Findings from 294 cases were categorized as follows: 87/294 (29.6%) patients had ground glass attenuations, 60/294 (20.4%) focal patchy shadows, 43/294 (14.6%) two-sided spotty shadows and 2/294 (0.7%) interstitial abnormalities. Chest CTs of four asymptomatic children revealed typical Covid-19 pneumonia imaging features [62]. Asymptomatic cases percentage is higher in children (7.6%) than in adults (1%) [122], highlighting the role of chest CT in early Covid-19 diagnostic work-up.

In a systematic review and meta-analysis including 9 pediatric Covid-19 case series, Chang et al. reported imaging features were analogous to those of adults [123]; the commonest being ground glass attenuations in 48% (95% CI 0.36–0.64; I2 = 5%, p = 0.52) and patchy consolidative opacities in 31% (95% CI 0.13–0.55; I2 = 51%, p = 0.09) of cases. 27% (95% CI 0.18–0.43; I2 = 0%, p = 0.64) of patients demonstrated no radiological abnormalities [124].

Covid-19 pneumonia's incidence in pediatric patients is low, clinical manifestations mild, course of disease curtailed and imaging features atypical in comparison with adults' forms potentially leading to misdiagnosis if solely depending on chest CT to screen infants. Posterior and peripheral lungs ground glass opacities appear less dense, limited in extent and may present in small nodular shapes [120,125,126]. Light forms are commoner in children and may present normal chest CTs [120,125,126]. Infrequently, disease progresses. Ground glass attenuations enlarge, increase in density, turn into multifocal consolidations and interstitial lesions become more apparent [126]. ‘White lung’ aspect rarely occurs [126]. In the recovery stage, complete lung abnormalities resolution takes place or merely slight linear attenuations persist [127]. Coinfections are usual in children rendering case preclusion difficult in the setting of determined epidemiological history and nonspecific CT features [120]. Numerous children exhibit pleural effusion [128]. Enlarged mediastinal lymph nodes were not reported. Scanning's indications careful weighting and low dose CT utilization are crucial to protect children from irradiation hazard. Chest CT supports diagnosis in considerably suspected patients with initially negative RT-PCR. Some RT-PCR positive children with initially normal CTs might develop anomalies subsequently. Since mild forms prevail in children, follow-up CT is only justifiable in the setting of clinical worsening and chest X-rays may represent a monitoring alternative [120,125,126]. In the absence of suggestive clinical manifestations and epidemiological history, chest radiography's usual indications are sustained (unexplained fever, abnormal pulmonary auscultation …) [129].

## 10 Imaging in pregnant women

Pregnant patients with confirmed Covid-19's imaging features are comparable to those of non-pregnant adults. In a study including 23 pregnant inpatients, chest CT showed subpleural ground glass attenuations, consolidative opacities, interstitial thickening, fibrous bands as well as concomitant pleural and/or pericardial effusions [95].

Elshafeey et al. systematic review included 385 pregnant women with Covid-19 pneumonia. Chest imaging informations were accessible for 125 patients (32.5%). 4 women (3.2%) had normal chest CTs. Bilateral anomalies were found in 99 patients (79.2%). Ground glass attenuations were noted in 81.6% (102), consolidative opacities in 17.6% (22), a reticular pattern in 0.8% (1), atelectasis in 0.8% (1), crazy paving in 0.8% (1), thickened pleura in 0.8% (1) and hydrothorax in 7.2% (9) of cases [130].

Pregnant women imaging's indications (chest x-ray and CT) should be carefully weighted to minimize irradiations' risks and patients well informed prior to their performance. Low-dose Ct scans are recommended [131], and fetus's local protection must be employed. The first trimester of pregnancy warrants special considerations for radiation's hazard. In this setting, proceeding to CT only when an initial chest x-ray is inconclusive is advisable [132].

## 11 Imaging of neurological manifestations

Poyiadji et al. reported the first presumed case of covid-19 related acute necrotizing hemorrhagic encephalopathy in a female patient admitted for cough, hyperthermia and mental status impairment whose nasopharyngeal swab RT-PCR was positive for Sars-CoV 2. Sars-Cov2 testing in the CSF was not performed. Brain CT demonstrated symmetrical bi-thalamic hypodensities with patent cerebral veins. Brain MRI revealed bilateral medial temporal lobes and thalami hemorrhagic lesions with ring of contrast enhancement [133].

In a correspondence to the new England journal of medicine, Helms et al. report neurologic manifestations in 58 patients admitted to Strasbourg intensive care units for covid-19 induced acute respiratory distress syndrome. 69% (40/58) were agitated, 67% (39/58) presented corticospinal tract signs and 36% (14/39) displayed a dysexecutive syndrome. Brain MRI was obtained in 13 cases and exhibited leptomeningeal enhancement in 62% (8/13), ischemic stroke in 23% (3/13) and perfusion anomalies in 100% (11/11) of patients [134].

Evidence determining whether these neurological features are specific to Sars-Cov2 infection or result from cytokine storm syndrome, severe disease-associated encephalopathy or drugs are still lacking [133,134]. In suspected or confirmed Covid-19 patients with neurological expressions; performing Brain MRI is encouraged.

## 12 Limitations

Our review has some limitations. The Covid-19 pandemic being a quickly evolving situation, rapid data sharing necessity could affect released reports’ quality. Despite new informations being published on a day-to-day basis rendering continual update an imperative, we think that major reported disease imaging findings will not be modified. Included studies were mainly case reports and case series limiting evidence certainty. Several reports were retrospective with a restricted sample size. Data accessibility was limited in certain instances. Larger cohorts and further longitudinal research are needed to clarify long-term follow-up outcomes and pulmonary sequelae. The correlation between imaging findings and anatomopathological pulmonary alterations are yet to be thoroughly investigated.

## 13 Recommendations

In asymptomatic patients, CT is not recommended as a Covid-19 screening test [79]. Nevertheless, when rapid PCR results are unavailable, chest CT can detect silent pulmonary lesions in emergency admissions with unidentified Covid-19 status whose conditions do not permit awaiting for biological tests results: urgent surgeries or therapeutic stances (stroke, bleeding). Imaging patients with mild Covid-19 symptoms is not indicated unless they have comorbidities (diabetes, chronic respiratory conditions and others more) [8,45]. In patients with moderate to severe Covid-19 features (dyspnea, desaturation) and regardless of tests results, CT is recommended to evaluate disease extent at baseline, help predict outcome and assisted ventilation requirements [45]. When initial RT-PCR tests are negative while CT findings indicate a Covid-19 pneumonia, testing is repeated to exclude false negatives [45]. A deteriorating respiratory status warrants a repeat CT [8,45]. Pulmonary embolism suspicions prompt contrast enhanced CT performance [45]. Reiterated CTs are not indicated in patients recovering from Covid-19 with no breathing impairment nor hypoxemia [8,45]. Repeat CTs indications should be cautiously weighted as Sars-Cov2 infected patients transportation from wards to radiology departments yields a high risk of contaminating other patients and health care workers [45]. Using standardized Covid-19 C T reporting language is recommended [79]. Chest radiography and lung ultrasound should not be used as a first-line screening or diagnostic test. Chest x-rays are used for non-transportable intensive-care-units patients follow-up while ultrasound is a bed-side tool that allows assisted ventilation parameters adjustment, induced complications diagnosis and fluid load surveillance [45].

## 14 Conclusion

In conclusion, this review offers an exhaustive analysis of the current literature on imaging role and findings in COVID-19 pneumonia. Chest CT plays an invaluable part notably in early disease detection in cases of high clinical suspicion and negative or inaccessible RT-PCR as well as pneumonia progression and treatment response evaluation. Guidelines and reporting templates provide a framework for radiologists to follow and better communication with clinicians. As this pandemic continues its rapid surge; artificial intelligence mediated imaging could strenghten available tools and help radiology staff.

## Ethical Approval

Ethical approval was not required.

## Consent

Consent for publication was not applicable.

## Author contribution

Hanae Ramdani and Nazik Allali: Contributed to conception, acquisition, analysis, and interpretation of data; drafted the manuscript. Siham el Haddad and Latifa Chat: Critically revised manuscript. All the authors have read and approved the final draft of the manuscript.

## Registration of Research Studies

1. Name of the registry:

2. Unique Identifying number or registration ID:

3. Hyperlink to your specific registration (must be publicly accessible and will be checked):

## Guarantor

Hanae Ramdani.

Email address: hanaeramdani@hotmail.fr.

## Consent for publication

Not applicable.

## Declaration of competing interest

Authors have no conflicts of interest.

No funding was received.
